# KaIDA: a modular tool for assisting image annotation in deep learning

**DOI:** 10.1515/jib-2022-0018

**Published:** 2022-08-26

**Authors:** Marcel P. Schilling, Svenja Schmelzer, Lukas Klinger, Markus Reischl

**Affiliations:** Institute for Automation and Applied Informatics, Karlsruhe Institute of Technology, D-76344 Eggenstein-Leopoldshafen, Germany

**Keywords:** data annotation, deep learning, deep neural networks, high-throughput screening, image processing

## Abstract

Deep learning models achieve high-quality results in image processing. However, to robustly optimize parameters of deep neural networks, large annotated datasets are needed. Image annotation is often performed manually by experts without a comprehensive tool for assistance which is time- consuming, burdensome, and not intuitive. Using the here presented modular Karlsruhe Image Data Annotation (KaIDA) tool, for the first time assisted annotation in various image processing tasks is possible to support users during this process. It aims to simplify annotation, increase user efficiency, enhance annotation quality, and provide additional useful annotation-related functionalities. KaIDA is available open-source at https://git.scc.kit.edu/sc1357/kaida.

## List of non-standard abbreviations

API: Application Programming Interface
DL: Deep Learning
DNN: Deep Neural Network
GUI: Graphical User Interface
KaIDA: Karlsruhe Image Data Annotation Tool
SPPO: Screening Platform for Personalized Oncology

## 1 Introduction

Deep Neural Networks (DNNs) often outperform traditional image processing methods [1] and are widely used in biomedical high-throughput screening applications, such as classification of tumors [2], instance segmentation of stained cell nuclei [3] to perform automated cell viability analysis as part of molecular biology experiments, semantic segmentation to analyze organs non-invasively [4], or seed detection for biodiversity studies [5]. The annotation of data by domain experts is crucial. However, it is time-consuming, expensive [1, 6], and demotivating. Often, annotation quality decreases over time and is inconsistent between annotators, which leads to noisy datasets [7].

There are software solutions to assist image annotation, though, their focus is mainly on the perspective of data scientists which hampers the application for domain-experts [8] such as biologists or clinicians (e.g. software/hardware requirements, source code instead of Graphical User Interfaces (GUIs)). Existing annotation tools [9–14] do not assist users during annotation at all, only provide partial assistance, or make it difficult to integrate methods for assistance. Thus, there is a lack of making annotation more enjoyable: Proposals for pre-annotation are missing to speed up the process, an automated selection of promising samples is not implemented, no automated annotation post-processing is available, there is no feedback regarding the quality of annotations made to keep annotators attentive, software is hard to handle without using up-to-date interfaces (GUI, touchscreen, pens etc.), and for each problem formulation (classification, detection, segmentation) different software exists. Further, although designing Deep Learning (DL) pipelines is an iterative process, concepts for versioning datasets are missing. Hence, neither a generic and comprehensive concept nor a corresponding open-source software implementation for assisted image annotation exists, although annotation impacts many projects since the potential of DL was discovered [15]. Furthermore, the research community lacks criteria for evaluating annotation tools and a detailed comparison of state-of-the-art tools, respectively.

Therefore, we contribute (i) a concept including an open-source software tool for assisted image annotation, (ii) introduce metrics for usability evaluation, and (iii) provide a scheme for comparing annotation tools. In addition, we demonstrate the application of our proposal, show its usability, and present the advantages compared to state-of-the-art tools.

## 2 Concept

### 2.1 Overview

**Figure 1:**
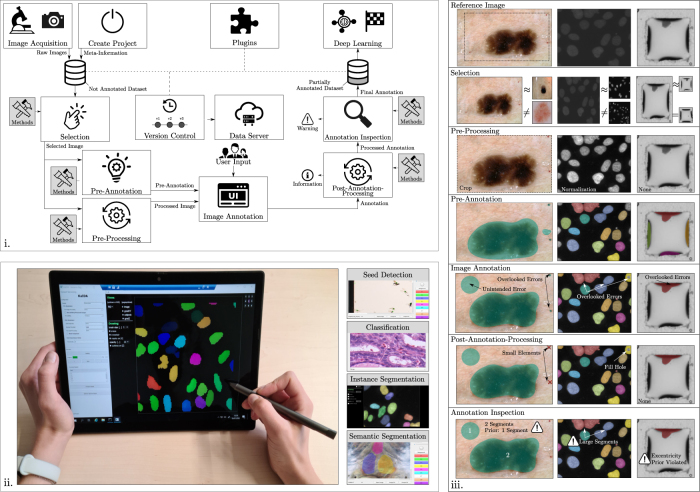
Overview KaIDA. The interaction of the individual modules is shown via an overview diagram. (ii) Application. A domain expert uses the tool KaIDA in the proposed setup (cf. Supplementary Information) for the annotation of images in the case of instance segmentation [3, 9]. In additioPopova2019FacileOneStepn, KaIDA supports seed detection, classification, and semantic segmentation [2, 4, 5]. (iii) Exemplary Results. Three reference images [3, 16, 17] are utilized to present the possible methods of KaIDA. Via “Selection” heterogeneous samples are obtained, “Pre-Processing” crops/normalize the image, and “Pre-Annotation” provides an initial annotation, which is adapted by a user during “Image Annotation”. Errors may remain or are introduced unintendedly. Via “Post-Annotation-Process” small noisy elements are removed/holes are filled and “Annotation Inspection” warns that the prior of one segment per image, average segment area, or excentricity of a given segment is violated. Additionally, it is shown that not every module needs to be activated (None).

We present the tool Karlsruhe Image Data Annotation (KaIDA) enabling various ways of assistance for annotating images, solutions for smart data management, and broader ideas to support the application of DL (cf. Figure 1  i). The proposal is based on Label Assistant [18], but extended to a generic and modular approach suited for various image processing tasks. The open-source software tool KaIDA supports the annotation of datasets, increases user efficiency, enhances annotation quality, manages dataset versions, and offers additional extensions useful in DL. Taking Figure 1  ii into account, we propose a practical setup of how KaIDA can be integrated into the workflows of future laboratories (cf. Supplementary Information). In Figure 1  iii, exemplary results during assisted image annotation are illustrated.

### 2.2 Modules

In the following, the modules are explained in detail. First, a raw dataset (“Image Acquisition”, obtained, i.e., by a scanner, microscope, camera) is supplied with meta-information (“Create Project”, e.g. class information or problem categorization) essential for scientific data handling [19].

#### 2.2.1 Selection

State-of-the-art open-source annotation tools do not support the idea of influencing the sampling order during annotation. To enable deep active learning [20], which has been considered primarily from a theoretical perspective, “Selection” allows the user to affect the order of images presented to focus on the most promising images instead of the naive sampling, i.e., from the first to the last image of a dataset.

KaIDA provides multiple selectors for affecting the order of images presented to users.

The state-of-the-art approach of sampling is depicted in the method of “Sequential Selection”, which sorts images in alphabetic order based on their corresponding file names.

The method “Cherry Picker” displays an additional user interface to allow a manual selection of samples that should be annotated first, e.g. a diverse subset of all images to train a generalizing DNN with less annotation effort [20].

In addition, using “Random Selection” favors increasing heterogeneity in datasets. The next sample is selected randomly, which is beneficial, especially, in sequential datasets including a large amount of similar images [18]. In general, this method is a good compromise in terms of computational effort and generalization performance of a DNN per used annotation.

Besides, we provide the method of “Heterogeneity Sampling” inspired by deep active learning [20]. First, a ResNet [21] decoder serves as a feature extractor to avoid the curse of dimensionality when comparing images. Due to using transfer learning on ImageNet, this method needs no additional training on the considered individual dataset, which is beneficial in terms of computational effort. Second, by using a similarity metric, i.e., cosine similarity or L2-norm, all images are compared pairwise. The sampling score solves an optimization problem to determine the most diverse sample given an already considered subset of images.

However, due to the large computational cost of doing inference w.r.t. all samples of the not annotated dataset, the sampling is saved in an external file. This is advantageous in two aspects: (i) in the case of continuing the annotation process, duplicate computation of scores is avoided, and (ii) sampling can be done on a more powerful device and imported only be by domain experts.

#### 2.2.2 Pre-processing

There are various scenarios where annotating raw images is challenging, i.e., sub-optimal imaging conditions, large images including irrelevant areas, or low pixel-to-object resolution. “Pre-Processing” is advantageous or even necessary for the annotation, but, ordinarily, separate tools such as Fiji/ImageJ [22] are utilized. KaIDA integrates pre-processing directly in the annotation pipeline and no separate tools are needed. We provide conventional image processing functions such as image normalization, noise filters, resampling to change the image resolution, or the creation of crops to focus only a region of interest given in an image.

#### 2.2.3 Pre-annotation

Frequently, prior knowledge w.r.t. image annotation exists, but is not used. Users start from scratch when annotating images. The concept of “Pre-Annotation” incorporates available algorithms to provide an initial annotation prediction [23] and is integrated into KaIDA. Only a correction of predicted annotations is required. The selection of a suited pre-annotation algorithm depends on the data.

For semantic segmentation, seed detection, and instance segmentation tasks, we provide traditional image processing algorithms such as thresholding based on Otsu, constant values, or percentiles in histograms of images. Additionally, it is possible to use the same annotation as for the previous image, which is especially useful for related/sequential image data (3D images or time series) when only small changes are assumed between images. In this case, using sequential selection is essential. Moreover, pre-trained DNNs are a method for pre-annotation. We provide the state-of-the-art method Cellpose [9] and an implementation of a U-Net [24]. Other custom architectures can be integrated into KaIDA due to our extendable concept (cf. Supplementary Information).

#### 2.2.4 Image annotation

The way of “Image Annotation” is not task-agnostic (e.g. pixel-wise annotations vs. annotation per image) whereby software tools often support only a specific image processing task. Hence, KaIDA adapts the input window [9, 11] for “User Input” considering the image processing task given in the meta-information to enable annotation in different image processing tasks.

#### 2.2.5 Post-annotation-processing

Practical projects show that some errors are reoccurring (e.g. holes in segments or unintended small noisy segments) [7, 25]. Post-processing of annotations is meaningful, but often not integrated into annotation tools. Using separate tools for post-processing is cumbersome and may be critical without a supervision by users. Hence, we integrate “Post-Annotation-Processing” to enhance the annotation quality. To ensure traceability, overlaying of raw and post-processed annotation is possible. Hence, the post-processing is monitored and unwanted changes are avoided. Morphological operators, a method to fill holes in segments, or to remove small elements are already implemented in KaIDA. Further, fuzzy annotation in the case of classification is conceivable.

#### 2.2.6 Annotation inspection

The work of Karimi et al. [7] addresses the issue of annotator variability leading to noisy annotations. There are methods to handle them [7, 25], i.e., using DNNs for the inspection of annotations, but currently, they are detached from the annotation process. An integration into state-of-the-art annotation tools lacks. KaIDA integrates an “Annotation Inspection” step, which evaluates annotations depending on suited quality criteria. The user selects thresholds to regulate warning appearances. In case of a triggered warning, the annotator is alerted and decides whether to re-inspect or keep the annotation.

Currently, region proposals can be used to inspect the quality of an annotation. For instance, annotators can use the criterion of area, the number of segments, or convexity based on prior knowledge. Furthermore, the idea of utilizing DNNs for inspection presented in [25] could be a further method. Annotations and predictions of uncertainty-aware DNNs are compared. However, it must be noted that using the same DNN in pre-annotation and annotation inspection is not meaningful, since a short circuit is formed in this scenario.

Finally, the annotation of the input image is obtained, the partially annotated dataset grows continuously, and investigations regarding supervised DL pipelines can be done.

#### 2.2.7 Version control

Annotating datasets is usually an iterative process. The required scope of annotated data is not known *a priori* [26] or a domain shift in images degrades DNN performance [27] and requires newly annotated samples. Besides, a changed annotation policy or correcting erroneous annotations are scenarios where the dataset changes within a project. Hence, datasets may develop during designing a DL processing pipeline, but tracking dataset versions in annotation tools lacks. We incorporate the ability of dataset “Version Control” using the ideas of [28] for handling large files via meta-files located in Git version control. Thus, KaIDA allows analyzing and tracking the history of datasets. Users can roll back to all available dataset versions. It is beneficial to track the changes over time, i.e., comparing DNN results using different dataset versions to check learning curves or the influence of a changing annotation policy. In addition, the dataset version control supports the usage of data servers, which is beneficial in several ways (transfer of data to computing clusters for DL training, data backup, or interaction of data scientists/domain experts).

#### 2.2.8 Plugins

Dealing with image annotation, additional features are helpful for users. Hence, we created an interface to hand over the data and functionalities of KaIDA to tool extensions referred to as “Plugins”.

Dealing with large high-resolution images, i.e., thousands of cell nuclei [3] or hundreds of insects, image cropping is meaningful in two aspects. First, annotating smaller images is more comfortable for users. Second, by using crops of images, the GPU memory requirements during training are reduced. However, the crop functionality is often done by a separate implementation and not integrated into state-of-the-art annotation tools. Hence, we offer an image crop plugin to create fragments of large images without the requirement of a separate solution.

It is difficult for domain experts to directly use standard DNN architectures such as U-Net [24] since they are only available as source code. An application plugin directly allows the usage of trained DNNs for individual projects to process the data of experimenters. Hence, the processing can be done by the domain experts themselves with no need for a request to data scientists regarding the processing. Though, DNN training or inference require high-performance computing resources, which are in the normal case not available for domain experts. By considering our proposal of the practical setup in Supplementary Information, a solution to solve this limitation is available. To process large-scale data, we support the integration of REST API in order to separate processing requests and the execution on powerful devices. State-of-the-art DNNs can be containerized (e.g. by using Docker images) and deployed on a high-performance computing cluster for the application. In this case, data needs only to be transmitted, but computation can be outsourced.

### 2.3 Software development and implementation

#### 2.3.1 General

KaIDA is available open-source for download/installation at https://git.scc.kit.edu/sc1357/kaida. State-of-the-art software tools are mainly suited for a special image processing and extensions are not intended. To overcome this issue, KaIDA has been developed on a modular level and is agnostic w.r.t. the underlying image processing task or the used methods in the modules. Since the forms of assistance are strongly related to the practical project, extensions or customization can be integrated into KaIDA due to its generic structure to ensure universal applicability. The current implementation supports the image processing tasks of (i) classification, (ii) semantic segmentation, (iii) instance segmentation, and (iv) seed detection, but an extension for other tasks is possible due to its generic software concept. Further information and details regarding the software development concept, the implementation, and its integration in future laboratories are given in Supplementary Information.

#### 2.3.2 Tutorial

**Figure 2:**
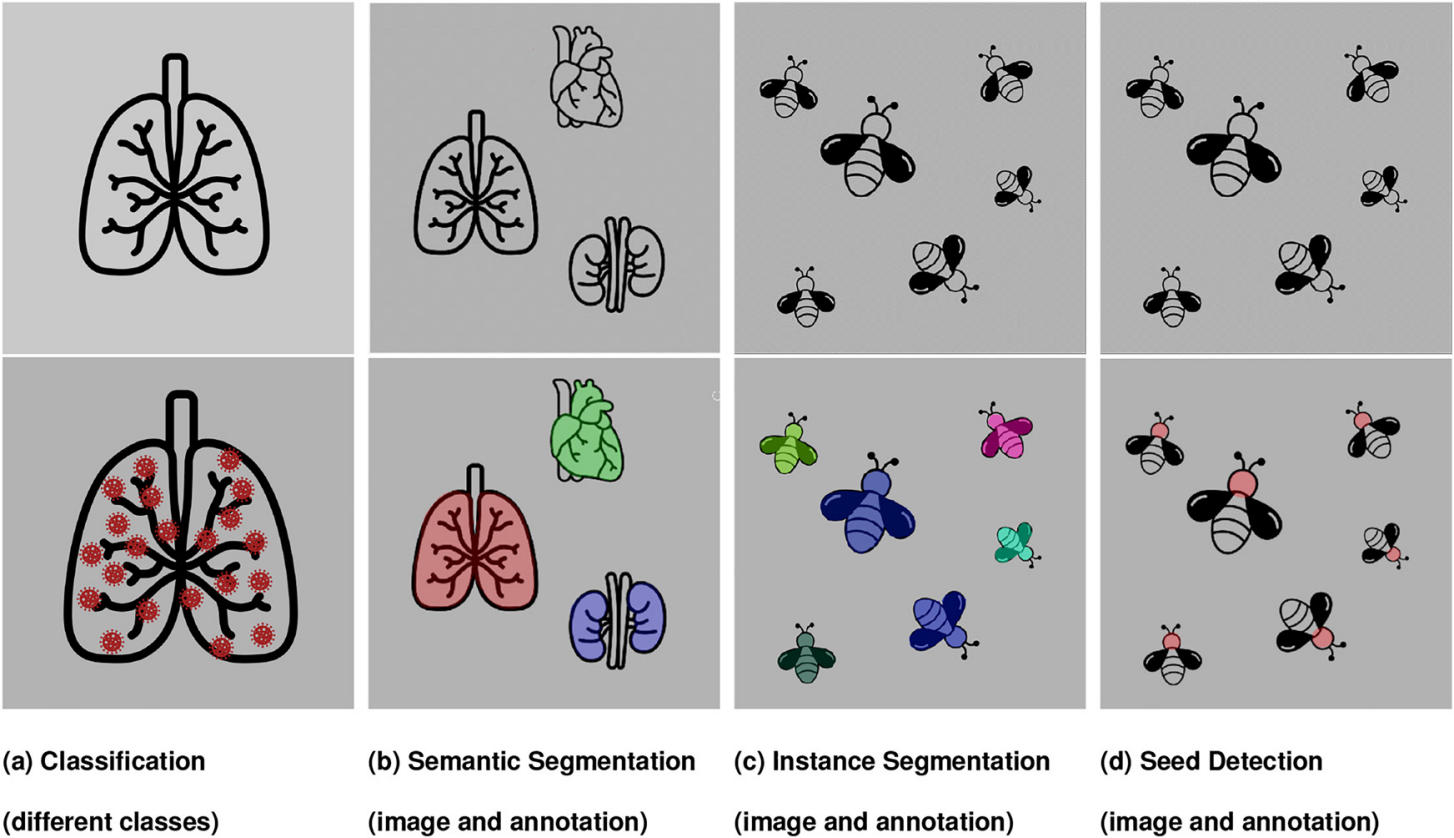
Overview tutorial datasets. Tutorial datasets are provided to facilitate the entry into the use of KaIDA. For instance, the classification (a) of not infected lungs (a, first row)/infected lungs (a, second row), the semantic segmentation (b) of organs (heart, lung, or kidney), and instance segmentation (c)/seed detection (d) of bees. The results of annotation regarding the tasks (b–d) are masks (second row). Whilst each color of the semantic mask represents a different class (b, second row), the colors of the mask resulting from instance segmentation represent a different instance of the same class (c, second row). In the case of seed detection, the intention is only to count instances and not to completely segment them. Hence, only the head of each bee is annotated (d, second row).

Taking software tools into account, uncomplicated operability for users with different backgrounds or levels of experience is important. To simplify the usage of KaIDA, we provide tutorial datasets for each image processing task. The dummy datasets are available at https://osf.io/5zcye/and given in Figure 2. Hence, users can test KaIDA and its functionalities directly. In addition, a manual to present the basic usage of KaIDA is presented in the open-source repository in form of a README file. However, the explanation of a tool by means of a video is often more beneficial for users. Thus, in addition, we provide a video per image processing tasks using the tutorial datasets to further lower the hurdles for new users for using KaIDA to annotate images. Thus, the whole workflow from initializing a new project to annotating exemplary samples is shown. All videos can be downloaded at https://osf.io/5zcye/.

#### 2.3.3 Usability

**Figure 3:**
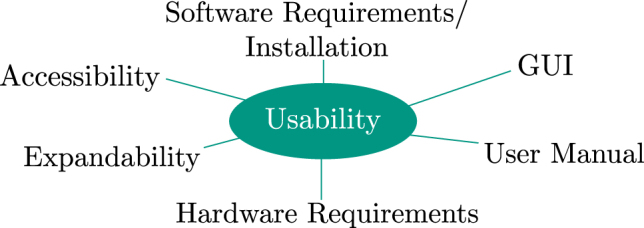
Criteria usability study. The authors of Grid Screener [29] enumerate accessibility, expandability, software/hardware requirements, an available GUI, and user manual as criteria for the evaluation of a software tool in terms of usability.

For proposing a software tool, it is important to consider and evaluate usability. We refer to the criteria introduced in the high-throughput image processing tool Grid Screener [29] to investigate the usability of KaIDA. The criteria are given in Figure 3. The authors name accessibility, and requirements regarding software and hardware as being relevant for the usability of a tool. Available user manuals and GUIs enhance the usage of tools. Further, modular expandability is beneficial for the application of tools in a broader sense, e.g. for other applications. An evaluation of KaIDA regarding those usability criteria is given in Section 3.2.

### 2.4 Scheme for comparing annotation tools

**Figure 4:**
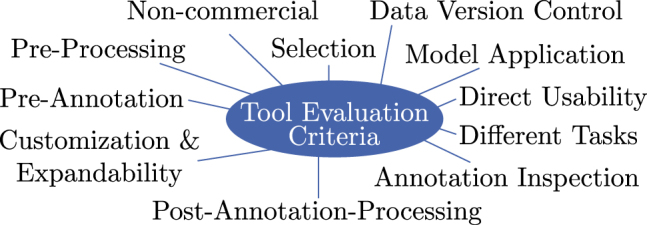
Scheme for comparing annotation tools. For the comparison of state-of-the-art image annotation tools, different criteria are used to consider different aspects.

A comparison of annotation tools is helpful for researchers in selecting an appropriate software tool for their DL project. Evaluation criteria are required for comparison. We consider different aspects in terms of functionality, methods, and usability. For clarity, we have summarized the criteria related to usability (cf. Section 2.3.3) to the ability towards customizing/extending a tool and the direct usability to start annotation with little effort when using a tool the first time. In addition, we consider whether the tool is non-commercial, supports different image processing tasks, provide a framework for training/inference step (cf. model application), and integrates data version control. Besides, the criteria that directly influence the process of image annotation are taken into account: functionality to influence the order of the presented samples (cf. selection), image pre-processing, pre-annotation, post-annotation-processing, and annotation inspection. Figure 4 presents an overview of all introduced criteria.

## 3 Application

### 3.1 Modules

To investigate our proposed concept, results of the introduced modules are investigated in the following. Since our contribution KaIDA is a software tool, this is done by using images from different application cases.

#### 3.1.1 Selection

**Figure 5:**
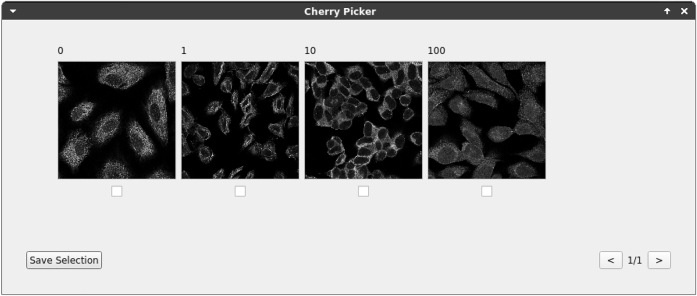
GUI cherry picker. KaIDA shows a GUI for cherry-picking images of the human brain atlas [30] via user input. The selected images would appear first during the annotation process.

Figure 5 illustrates the method “Cherry Picker” using the tutorial dataset. Users can select the most promising samples via a GUI. Those images would be presented to the annotator first during image annotation.

**Figure 6:**
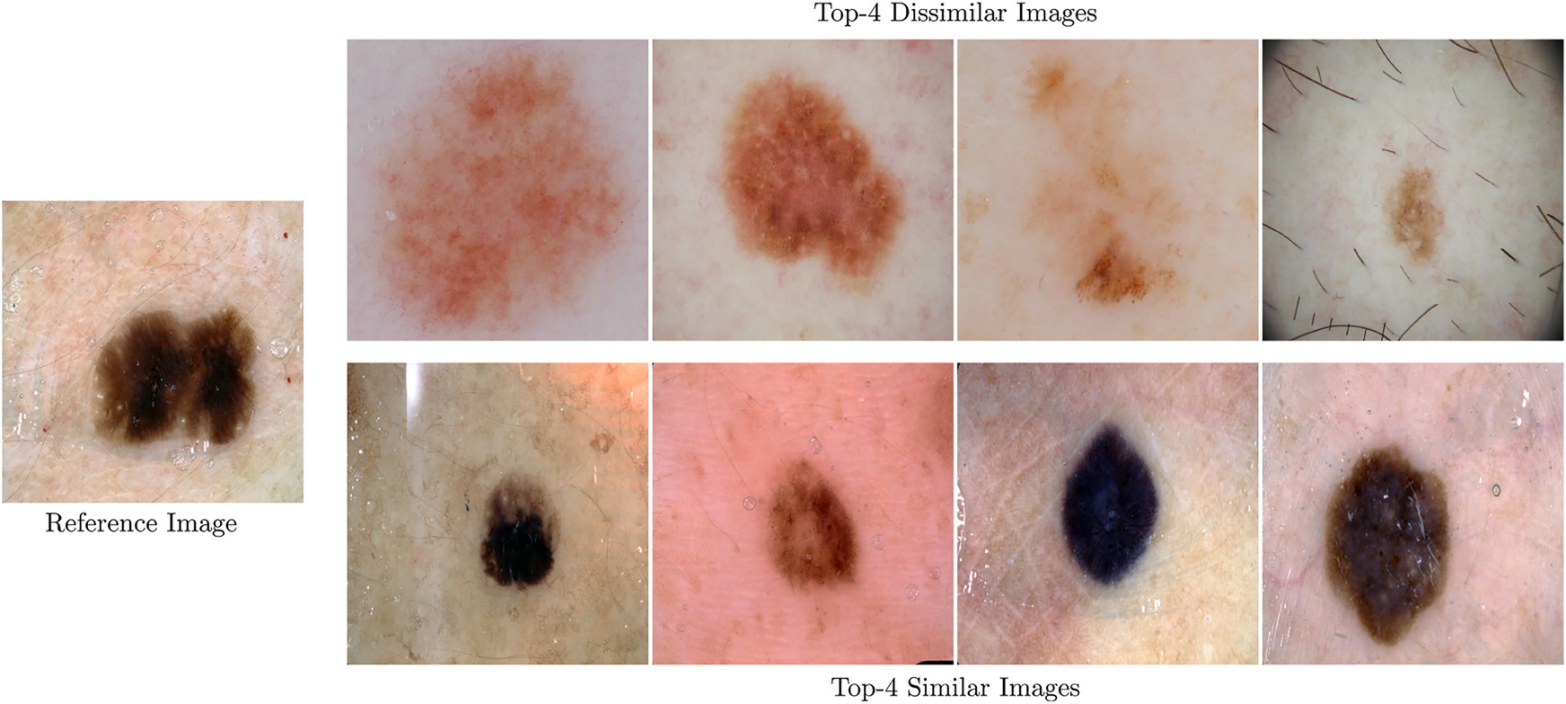
Visual validation heterogeneity sampling. Exemplary results of the heterogeneity sampler are presented. Considering a reference image of the ISIC 2017 Melanoma image segmentation dataset [16], the top-4 similar and top-4 dissimilar images are opposed. It is visible that the sampler is capable of distinguishing similar and dissimilar images using a pre-trained ResNet [21] decoder for feature extraction.

Furthermore, the results of “Heterogeneity Sampling” are taken into consideration. Figure 6 illustrates exemplary results of obtaining similar and dissimilar images given a reference image w.r.t. the ISIC 2017 Melanoma image segmentation dataset [16]. A visual comparison shows that using ResNet [21] for feature extraction is a feasible way to compare images regarding similarity.

#### 3.1.2 Pre-processing

**Figure 7:**
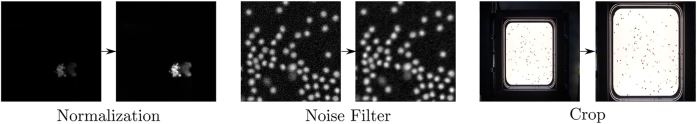
Illustration of methods in pre-processing. Exemplary results of normalization, noise filter, or crop are presented.

To present the functionality of pre-processing, examples are given in Figure 7. It shows impressively the advantage of pre-processing. Using image normalization, noise filtering, or extracted crops simplifies the annotation for users.

#### 3.1.3 Pre-annotation

**Figure 8:**
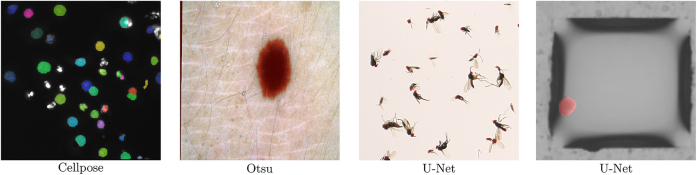
Illustration of methods in pre-annotation. Exemplary [3, 5, 16, 17] results of pre-annotations computed by Cellpose [9], Otsu, and U-Net [24]. Few errors remain in the predictions, but the visualization clearly shows that correcting annotations is easier than annotating from scratch.

Figure 8 illustrates examples of different pre-annotation methods already implemented in KaIDA. The pre-annotations are not correct in total. Nevertheless, the advantage of pre-annotation becomes clear, as it is easier to correct annotations than to start from scratch, i.e., dealing with numerous cell instances.

#### 3.1.4 Post-annotation-processing

**Figure 9:**
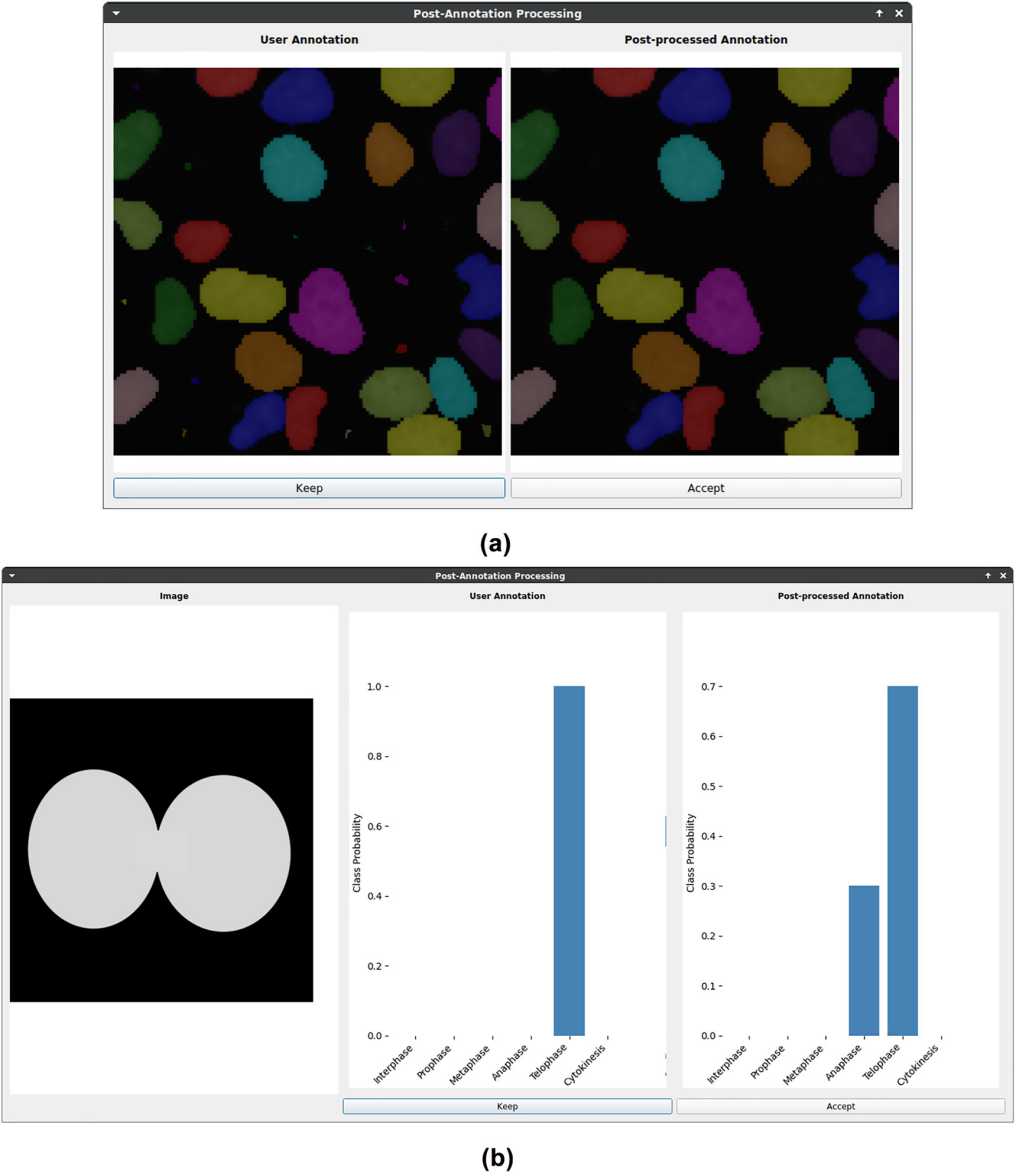
Illustration of methods in post-annotation-processing. Exemplary results of post-annotation processing. (a) Instance Segmentation. The method of removing small objects is displayed. (b) Classification. The exemplary method for fuzzy annotation is shown for a synthetic image illustrating cell mitosis.The sharp label of telophase is transformed into a mixture of telophase and anaphase. Hence, fuzzy annotation can beused in ambiguous cases.

The corresponding GUI of “Post-Annotation-Processing” is depicted in Figure 9. A comparison between user annotation and post-processed annotation is displayed to ensure traceability. The user can choose which one to keep. We present an example of removing small objects in the case of instance segmentation (cf. Figure 9a). Further, fuzzy annotation in classification tasks is shown in Figure 9b.

#### 3.1.5 Annotation inspection

**Figure 10:**
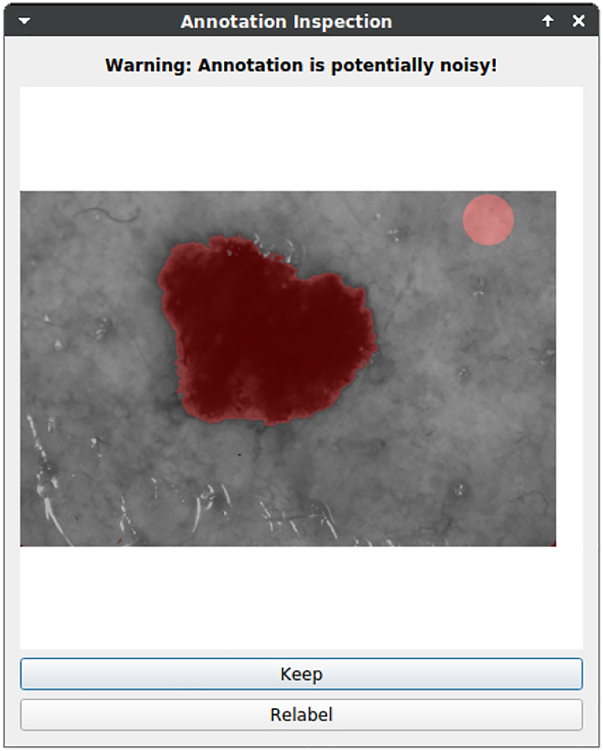
Illustration of methods in annotation inspection. Exemplary results of using the number of segments as an annotation quality criterion. Assuming only one melanoma per image, a warning is shown because two segments are marked.

Figure 10 shows a scenario in which an inspection warning is triggered considering melanoma segmentation. It is assumed that only a single segment is visible per image. Since there are two marked segments, the user can choose to keep the annotation or to re-label the sample.

#### 3.1.6 Version control

**Figure 11:**
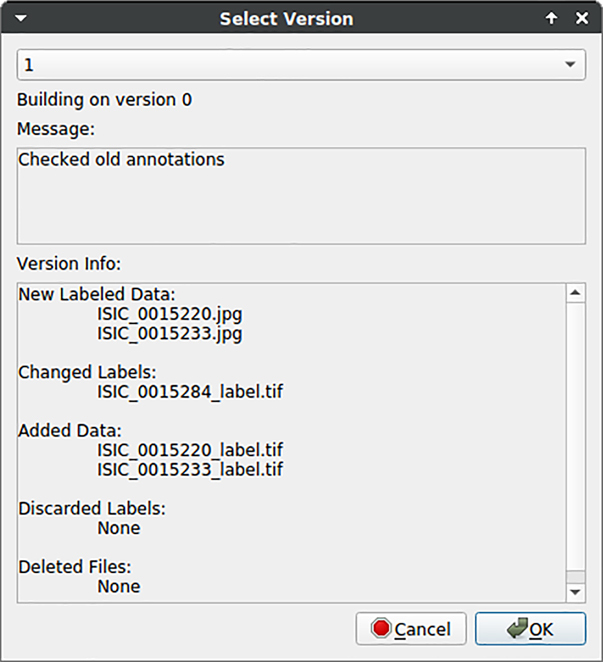
Feedback of data version control to users. Changes in the dataset are listed to give feedback to users regarding different dataset versions.

Figure 11 illustrates the GUI in the context of “Version Control”. Users receive feedback in terms of changes between different dataset version and may check out them in the current working directory.

#### 3.1.7 Plugins

**Figure 12:**
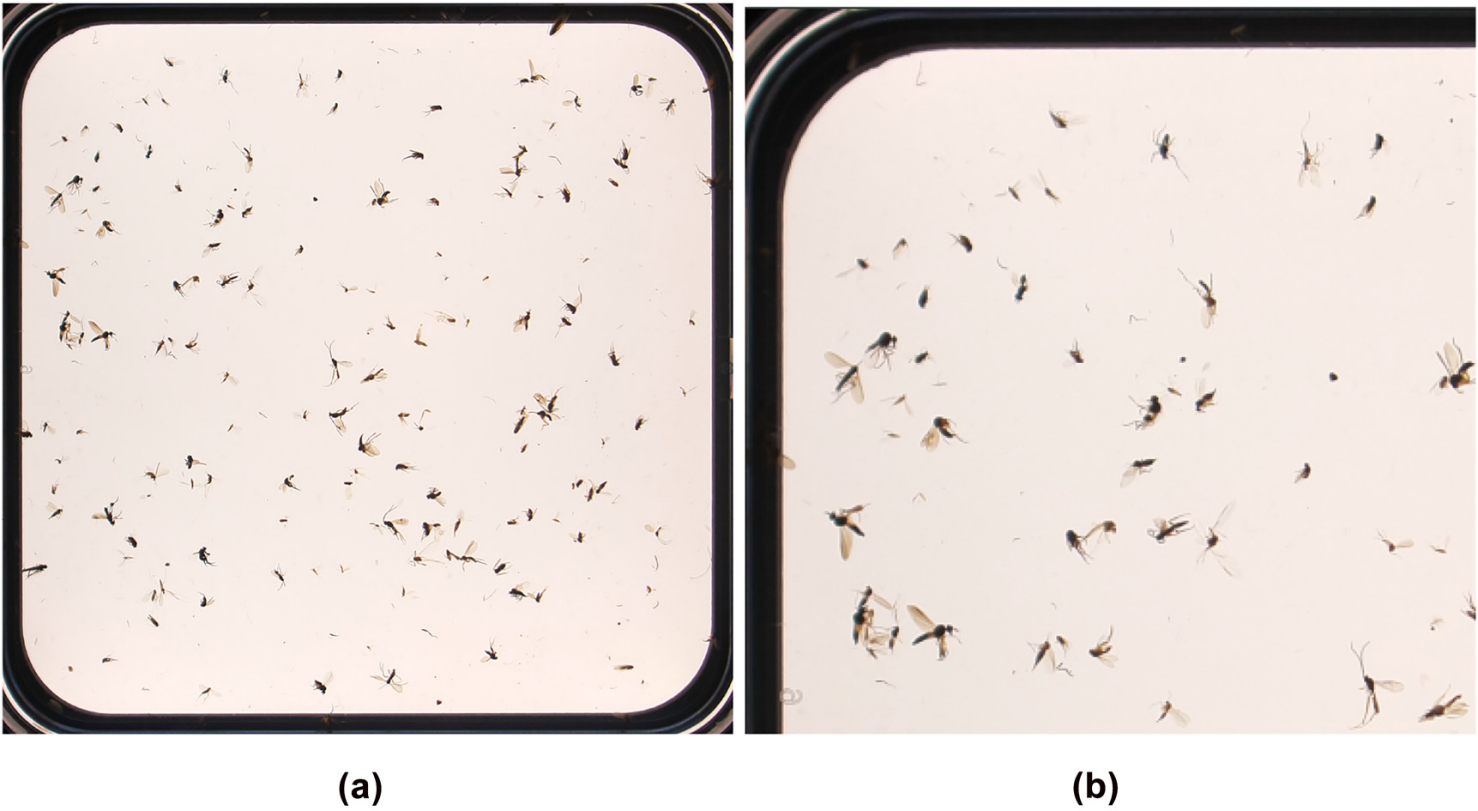
Illustration of image cropping. A crop (b) of an original image (a) is generated [5]. Using this example, it becomes clear that generating crops can simplify annotation in the case of many instances given within a sample. (a) Original Image. (b) Cropped Image.

Taking plugins into account, the benefit of generating image crops is demonstrated in Figure 12. Comparing the original image (a) and the image crop (b), annotators benefit from increased clarity within a considered crop in the case of many visible instances.

**Figure 13:**
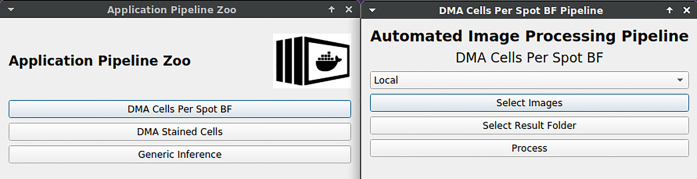
DNN application. User interface is shown for submitting a job to a computing server via REST API in order to apply image processing pipelines including a DNN.

KaIDA supports the integration of requests via REST API. Figure 13 illustrates an interface for the submission of a computation job via web requests nested in KaIDA. This allows the domain expert to apply trained DNNs/entire image processing pipelines directly and take the advantage of computing clusters. Thereby, it is only necessary to select the files intended for processing.

### 3.2 Usability

The results of the usability study are shown in Table 1. KaIDA is available as an open-source project in a public repository which leads to full target achievement w.r.t. accessibility. By providing a user manual in form of a README file, tutorial datasets, and corresponding tutorial videos, the criterion of an available user manual is fulfilled completely. Furthermore, we provide a GUI to enhance the usage of our proposed tool. Deploying the software in python as pip package and providing the corresponding software dependencies via a conda environment, software requirements are low and installation can be done comfortably by users. However, basic computer skills are required leading to a ranking of partial target achievement. To assist in less computing time, a GPU or high-performance CPU is advantageous. However, KaIDA can be used by a non-powerful CPU as well, yet, leading to longer computational time during assistance depending on the used methods. Thus, the criterion of low hardware requirements is not fulfilled completely. By using the comprehensive practical setup for future laboratories presented in Supplementary Information or REST API approaches, the previously mentioned objections, which lead to no full target achievement, can be eliminated. Thus, full target achievement is possible. As depicted in Section 2.3, KaIDA focuses on the objective of a modular and generic tool. Users can customize and extend the software tool since we provide the open-source repository including explanations regarding ways of extension. However, the users need basic coding knowledge in python. Therefore, no full target achievement in terms of expandability can be held.

**Table 1:** Usability study. The introduced criteria in grid screener [29] are evaluated. Thereby, “*✓*” indicates full, “(*✓*)” partial, and “*x*” no target achievement.

Criterion	Evaluation result
Accessibility	*✓*
User manual	*✓*
GUI	*✓*
Software requirements/Installation	(*✓*)
Hardware requirements	(*✓*)
Expandability	(*✓*)

In total, we can demonstrate the usability of KaIDA for researchers from various backgrounds. The hypothesis is supported by fully satisfying three criteria (five criteria using the proposal given in Supplementary Information or computation via REST API) and partially satisfying three criteria (one criterion following the concept in Supplementary Information/REST API approach).

### 3.3 Comparison of annotation tools

Taking the introduced criteria in Section 2.4 into account, we provide a comprehensive comparison of the state-of-the art image annotation tools, i.e., Image Labeling Tool [11], labelMe [13], LabelImg [12], Cellpose [9], CVAT [10], hasty.ai [14], and our proposal KaIDA. The result are given in Table 2 and are presented in detail below.

**Table 2:** Comparison of image annotation tools. A detailed comparison of different image annotation tools w.r.t. the introduced evaluation criteria is presented. Used notation: “*✓*” full, “(*✓*)” partial, and “*x*” no target attainment.

Criterion	Tools						
	**Image Labeling Tool** [11]	**labelMe** [13]	**LabelImg** [12]	**Cellpose** [9]	**CVAT** [10]	**hasty**.**ai** [14]	**KaIDA** (**Ours**)
Selection	*x*	*x*	*x*	*x*	*x*	*x*	*✓*
Pre-annotation	*x*	*x*	*x*	*✓*	*✓*	*✓*	*✓*
Pre-processing	*x*	*x*	*x*	*x*	*✓*	*x*	*✓*
Post-annotation-processing	*x*	*x*	*x*	*x*	*x*	*x*	*✓*
Annotation inspection	*x*	*x*	(*✓*)	*x*	(*✓*)	*✓*	*✓*
Different tasks	*x*	*✓*	*x*	(*✓*)	*✓*	*✓*	*✓*
Customization & expandability	*x*	*x*	*x*	*x*	(*✓*)	(*✓*)	*✓*
Data version control	*x*	*x*	*x*	*x*	*x*	*x*	*✓*
Non-commercial	*✓*	*✓*	*✓*	*✓*	*✓*	*x*	*✓*
Model application	*x*	*x*	*x*	(*✓*)	*✓*	*✓*	*✓*
Direct usability	*✓*	*✓*	(*✓*)	(*✓*)	*✓*	*✓*	(*✓*)

#### 3.3.1 Selection

KaIDA is the only tool that allows a change the order of sampling during annotation to focus on the most relevant samples given a limited time budget.

#### 3.3.2 Pre-annotation

The lightweight tools Image Labeling Tool, labelMe, LabelImg do not support pre-annotation natively. In contrast, the other annotation tools (Cellpose, CVAT, hasty.ai, KaIDA) allow the integration of functions to provide users with inital annotations.

#### 3.3.3 Pre-processing

CVAT offers an integration of OpenCV for the pre-processing of images. In parallel, KaIDA enables the pre-processing of raw images. The other tools take image pre-processing not into account.

#### 3.3.4 Post-annotation-processing

The idea of directly post-processing annotation based on prior knowledge is a concept unique to KaiDA.

#### 3.3.5 Annotation inspection

CVAT supports the idea of manually reviewing annotations. LabelImg integrates a functionality to mark inspected annotations with a flag. However, automated annotation inspection approaches are only given in hasty.ai and KaIDA.

#### 3.3.6 Different tasks

Only Image Labeling Tool (semantic segmentation) and LabelImg (object detection) focus on a single task. Cellpose partially meets the criterion as it only considers instance segmentation but in 2D and 3D modality. The other tools support different image processing tasks.

#### 3.3.7 Customization & expandability

CVAT allows to integrate user-defined models for pre-annotation. Further, hasty.ai offers an Application Programming Interface (API) which allows to customize parts of the elements, i.e., switching to a local custom model for pre-annotation. However, KaIDA is the only tools which focus on customization and expandability of methods in every module leading to more flexibility.

#### 3.3.8 Data version control

The concept of data version control is only available in KaIDA.

#### 3.3.9 Non-commercial

Except hasty.ai, all other tools are non-commercial.

#### 3.3.10 Model application

Lightweight tools such as Image Labeling Tool, labelMe, LabelImg are not designed to directly generate a model using the annotated dataset. Cellpose does not support the training of DNNs via a GUI, but provides an API to train and use DNNs. CVAT, hasty.ai, and KaIDA integrate a functionality for application of DL.

#### 3.3.11 Direct usability

Image Labeling Tool and labelMe provide executables for various operating systems. Partially target fulfillment can only be stated in the case of LabelImg (executable only for Windows, manual installation for others) and Cellpose/KaIDA (manual installation). CVAT and hasty.ai can be used directly thanks to a web interface.

## 4 Discussion

The results given in Section 3 show that there are several ways to expand the state-of-the-art annotation process. We present the functionalities in terms of different example dataset. Improvement and benefits regarding efficiency and quality of annotations for annotators by using KaIDA are demonstrated in contrast to the state of the art “no support”. By using practical examples, the relevance of presented methods and their applicability for users is demonstrated.

In addition, the results show that the support strongly depends on the particular dataset, which means that the methods cannot be used in every project. The ability to disable modules or extend them with custom implementations can address this issue. However, basic coding knowledge is required to integrate custom methods. Further, when using elaborate methods in the assisted annotation process, powerful computers are required to avoid long computation times if the proposed setup (cf. Supporting Information) is not considered.

By comparing KaIDA with state-of-the-art image annotation tools, we show that KaIDA bundles various aspects of annotation improvement. Most state-of-the-art tools only consider parts of the assistance portfolio and/or are commercial. However, a limitation of KaIDA is the lack of a web interface/executable to further reduce the installation effort if the suggested practical server setup (cf. Supporting Information) is not used.

## 5 Conclusions

With KaIDA, we contribute to the research community a ready-for-use software tool for assisted image annotation. The advantages show up in (i) efficient, simplified, and high-quality annotations, (ii) customization/expandability, and (iii) additional features. The enhancement of image annotation boosts supervised DL approaches in biomedical image processing, but is not limited to this case of application. Current research considers implementation of further methods per module in KaIDA as well as the integration towards 3D image annotation. Moreover, the creation of a web interface/executable is currently in preparation.

## Supplementary Material

Supplementary Material Details
