# Supplementary material for: KaIDA: a modular tool for assisting image annotation in deep learning
Source: J Integr Bioinform. 2022 Aug 26;19(4):20220018. doi: 10.1515/jib-2022-0018 (PMC9800041; doi:10.1515/jib-2022-0018)
Supplement: Supplementary file 1 — Supplementary Material Details [file j_jib-2022-0018_suppl.pdf]

KaIDA: A Modular Tool for Assisting Image Annotation in Deep Learning - Supplementary Information -

Marcel P. Schilling*, Svenja Schmelzer, Lukas Klinger, and Markus Reischl

Institute for Automation and Applied Informatics, Karlsruhe Institute of Technology, Eggenstein-Leopoldshafen, 76344, Germany.

*marcel.schilling@kit.edu

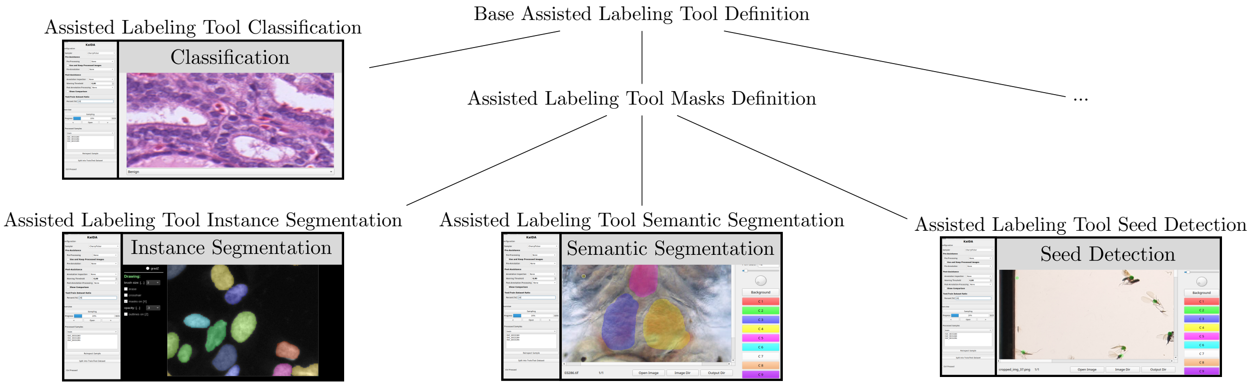

(a) Structure Tool. The individual task-specific tool is formed of a base definition.

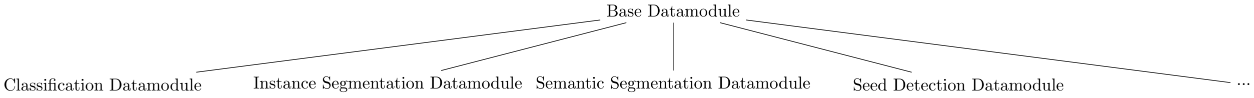

(b) Structure Datamodule. The datamodule used for each task is a specification of a defined base datamodule.

Figure 1: Extendable Software Concept of KaIDA. The general concept of KaIDA is following the concept of inheritance. A base element (parent) is defined and specified for the individual tasks (child), i.e., in the case of the whole tool (a) or elements such as the datamodule (b) implementation.

Implementation

General

Common problems dealing with software tools suffer from a lack of open-source accessibility, a difficult installation process, or the availability only for one specific operating system. With a user manual in the form of a README located in an open-source repository (`https://git.scc.kit.edu/sc1357/kaida`), researchers are guided in the installation and usage of KaIDA. By developing KaIDA as a `pip` package and including the corresponding dependencies in terms of a `conda` environment, the tool can be installed easily on users' devices by following the manual without hurdles. We have tested the cross-platform software package on different operating systems (Windows 10, Ubuntu 20.04) using python 3.8.5. All requirements and dependencies are listed in the open-source repository.

Extendable Software Development Concept

Frequently, tools are designed either adaptable, but not usable for non-developers or easy to use but not adaptable.

A major strength of our tool is its scope of application in the field of domain experts and computer scientists as well. The provided standard functionalities and GUI enables the direct usage for domain experts. Besides, an important objective in the design of KaIDA is providing a tool that is modular and generic to allow adaptations.

This flexibility can be understood on two levels, namely regarding image processing tasks and methods given in modules for an individual task. Therefore, we designed our concept initially task-independent to guarantee a maximum level of flexibility for the application.

Using interchangeable GUIs [1, 4] for annotation and applying inheritance approaches during software development, additional tasks can be added without hurdles by developers. Figure 1 presents the structure used to achieve the objective of providing an extendable tool. Strictly following the software development concept of inheritance, basic architectures are defined as parents. The individual task (child) is formed as a specification of the basic definition (parent). Hence, the clarity and extendibility of code are supported by this idea. We natively support four image processing tasks.

Considering assistance in image annotation, methods for assistance are various and related to the underlying task or dataset. Analogously to the explained concept of inheritance regarding the tool/datamodule, our proposed concept of extendibility w.r.t. modules is given in Figure 2. Defining an abstract module (parent), the basic structure including definitions of interfaces is provided for developing individual methods available

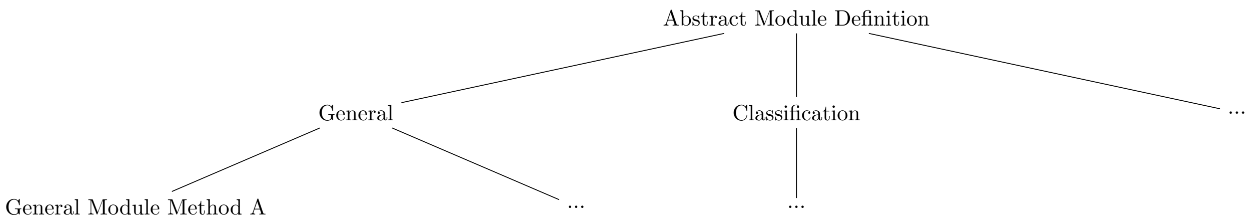

Figure 2: Extendable Software Concept Modules in KaIDA. Considering the idea of inheritance, an abstract module definition (parent) serves as a backbone for the individual methods (child). Further, methods are separated in the categories of general and task-related.

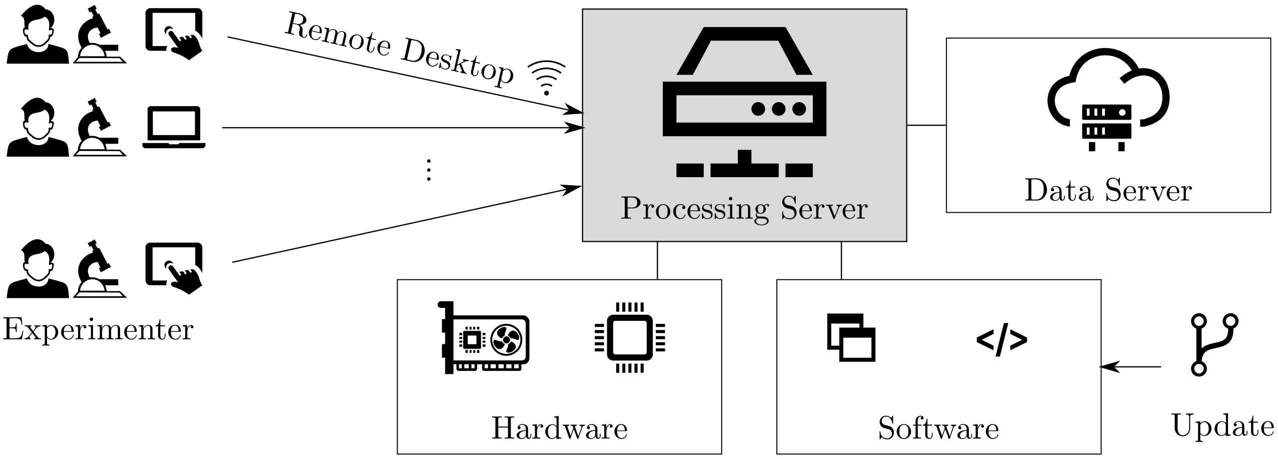

Figure 3: Practical Setup. The experimenter can use a remote desktop to connect individual devices (e.g. tablet, desktop computer, or mobile computer) with the processing server. Thereby, powerful hardware (GPU + CPU) and needed software are established to guarantee a comfortable and time-efficient usage of the KaIDA functionalities. An interface for software updates is integrated in the concept. Data transfer is enabled using a server connection.

in the corresponding module. The methods are clustered in general and task-related categories, respectively. Taking meta-information of the underlying task into account, KaIDA loads both, general and corresponding task-related methods automatically. We already provide various methods for each natively supported image processing task which are explained in the manuscript.

Coding details regarding both ways of extension are depicted in the open-source repository.

To sum up, KaIDA can be extended for the usage of other image annotation tasks (e.g. classification of 3D image stacks) and customized by adding new methods of a specific module (e.g. a new selection strategy or pre-annotation algorithm). This feature is of particular interest from the perspective of computer scientists. Hence, the deployment of newly developed methods to users needs no great effort.

Practical Setup

Concept

The usage of tools may be burdensome for domain experts due to high software/hardware requirements [2]. Hence, we take one step further and present a practical setup to scale the application of KaIDA in a broader context. Figure 3 illustrates the proposed concept. Experimenters can utilize remote desktop for establishing a connection to the processing server on their device (e.g. tablet, desktop computer, or mobile computer). This idea is advantageous from various points of view. First, our concept is independent regarding devices. Second, remote desktop is available on various operating systems (MacOS, Windows, Linux). Third, devices such as tablets can be used, which enhance pixel-wise image annotation for users. Further, a central processing server established with powerful hardware composed of GPU and CPU reduces the computational time during assisted annotation without imposing local requirements on the domain experts' devices. Besides, the establishment of all software needed guarantees a comfortable usage of KaIDA without the requirement of additional installations. A connection for software updates needs to be considered for the dynamic development

Table 1: Details w.r.t. established prototype setup.

Elements	Description
Processing Server	Operating System: Windows Server 2019
CPU	Intel Xeon 4210R
GPU	Nvidia Quadro RTX 4000 (8 GB)
Local Storage	RAM: 32 GB, 512 GB SSD
Device for Experimenter	Lenovo x12 Detachable (borrowable)
Software	`miniconda`
Update	Git Version Control
Data Server	LSDF

in DL projects to deploy new features such as developed assistance methods easily. In addition, the required data transfer is realized by a connection to a shared data server.

Prototype

We have realized a prototype construction regarding our proposed concept given in Table 1. The processing server uses Windows Server 2019 to enable remote desktop connections. The hardware setup is composed of an Intel Xeon 4210R CPU, Nvidia Quadro RTX 4000 GPU, 32 GB RAM, and 512 GB for local temporary storage. We offer the option to borrow a Lenovo x12 Detachable for using a touchscreen for image annotation. Git is used for version control and software updates. Further, the Large Scale Data Facility (LSDF) [3] is considered as a data server.

Supplementary Data

- Tutorial Datasets (`https://osf.io/5zcye/`)
- Video: Usage of KaIDA (`https://osf.io/5zcye/`)
- README/Manual KaIDA (`https://git.scc.kit.edu/sc1357/kaida`)
- Code (`https://git.scc.kit.edu/sc1357/kaida`)

References

[1] Andreas Bartschat. *Image Labeling Tool.* Accessed: 2021-10-21. [Online]. Available: `https://bitbucket.org/abartschat/imagelabelingtool`. 2019.

[2] Estibaliz Gómez-de-Mariscal et al. "DeepImageJ: A User-Friendly Environment to Run Deep Learning Models in ImageJ". In: *Nature Methods* 18.10 (2021), pp. 1192–1195.

[3] Thomas Jejkal et al. "LAMBDA – the LSDF Execution Framework for Data Intensive Applications". In: *Euromicro International Conference on Parallel, Distributed and Network-Based Processing.* 2012, pp. 213–220.

[4] Carsen Stringer et al. "Cellpose: A Generalist Algorithm for Cellular Segmentation". In: *Nature Methods* 18.1 (2021), pp. 100–106.
